# Humanin G (HNG) protects age-related macular degeneration (AMD) transmitochondrial ARPE-19 cybrids from mitochondrial and cellular damage

**DOI:** 10.1038/cddis.2017.348

**Published:** 2017-07-20

**Authors:** Sonali Nashine, Pinchas Cohen, Marilyn Chwa, Stephanie Lu, Anthony B Nesburn, Baruch D Kuppermann, M Cristina Kenney

**Affiliations:** 1 Department of Ophthalmology, Gavin Herbert Eye Institute, University of California Irvine, Irvine, CA, USA; 2 Davis School of Gerontology, University of Southern California, Los Angeles, CA, USA; 3 VA Medical Center Long Beach Hospital, Long Beach, CA, USA; 4 Cedars-Sinai Medical Center, Los Angeles, CA, USA; 5 Department of Pathology and Laboratory Medicine, University of California Irvine, Irvine, CA, USA

## Abstract

Age-related macular degeneration (AMD) ranks third among the leading causes of visual impairment with a blindness prevalence rate of 8.7%. Despite several treatment regimens, such as anti-angiogenic drugs, laser therapy, and vitamin supplementation, being available for wet AMD, to date there are no FDA-approved therapies for dry AMD. Substantial evidence implicates mitochondrial damage and retinal pigment epithelium (RPE) cell death in the pathogenesis of AMD. However, the effects of AMD mitochondria and Humanin G (HNG), a more potent variant of the mitochondrial-derived peptide (MDP) Humanin, on retinal cell survival have not been elucidated. In this study, we characterized mitochondrial and cellular damage in transmitochondrial cybrid cell lines that contain identical nuclei but possess mitochondria from either AMD or age-matched normal (Older-normal (NL)) subjects. AMD cybrids showed (1) reduced levels of cell viability, lower mtDNA copy numbers, and downregulation of mitochondrial replication/transcription genes and antioxidant enzyme genes; and (2) elevated levels of genes related to apoptosis, autophagy and ER-stress along with increased mtDNA fragmentation and higher susceptibility to amyloid-*β*-induced toxicity compared to NL cybrids. In AMD cybrids, HNG protected the AMD mitochondria, reduced pro-apoptosis gene and protein levels, upregulated gp130 (a component of the HN receptor complex), and increased the protection against amyloid-*β*-induced damage. In summary, in cybrids, damaged AMD mitochondria mediate cell death that can be reversed by HNG treatment. Our results also provide evidence of Humanin playing a pivotal role in protecting cells with AMD mitochondria. In the future, it may be possible that AMD patient’s blood samples containing damaged mitochondria may be useful as biomarkers for this condition. In conclusion, HNG may be a potential therapeutic target for treatment of dry AMD, a debilitating eye disease that currently has no available treatment. Further studies are needed to establish HNG as a viable mitochondria-targeting therapy for dry AMD.

Age-related macular degeneration (AMD), a devastating retinal disease, is a leading cause of irreversible blindness worldwide.^1^ Pathologically, early AMD manifests with formation of drusen deposits between the retinal pigment epithelium (RPE) and Bruch’s membrane. Advanced stages manifest with significant RPE degeneration, along with photoreceptor loss (geographic atrophy), and/or choroidal neovascularization.^2^

Several effective anti-VEGF drugs are available to treat the wet form of AMD.^3^ For the dry form of AMD, administration of various antioxidant formulations is recommended but there are no FDA-approved drugs.^4^

Retina is one of the highest energy demanding and oxygen-consuming tissues of the human body, relying on oxidative mitochondrial metabolism for ATP formation.^5^ Mitochondrial DNA (mtDNA) is a 16.5 kb, circular, double-stranded molecule comprised of 37 genes that code for 2 ribosomal RNAs, 22 transfer RNAs, and 13 proteins, which play key roles in the electron transport chain and oxidative phosphorylation.^6^ mtDNA is particularly susceptible to oxidative damage because of its poor DNA repair capacity. Any mtDNA damage causes perturbations in energy metabolism, leading to oxidative stress, depletion of antioxidants and eventual RPE cell death. A large body of literature has linked mitochondrial dysfunction and resulting RPE cell death to the development and progression of AMD, glaucoma, diabetic retinopathy.^7, 8, 9, 10, 11, 12^

Recent studies have identified mitochondrial-derived peptides (MDPs) that are coded from the mtDNA.^13, 14, 15^ The first MDP, Humanin, is a 24-amino acid polypeptide that was discovered while screening cDNA libraries from brain of a patient with Familial Alzheimer’s Disease. Humanin has a molecular weight of 2687.26 Da and is encoded from a small open reading frame within the *16S* ribosomal RNA gene, *MT-RNR2,* of the mitochondrial genome.^16^ Humanin is cytoprotective against amyloid-*β* induced toxicity in neuronal cells both *in vitro* and *in vivo*,^17, 18^ against cerebral ischemia and cardiac damage in mouse models,^19, 20^ and in numerous neurodegenerative disease models for Alzheimer’s disease, Huntington’s disease, and Prion diseases.^21, 22, 23^ Moreover, Humanin rescues primary RPE cells from oxidative damage and subsequent death *in vitro*.^24^ Each of the 24 amino acids in the Humanin peptide have a specific function. Serine at position 14 confers neuroprotection,^25^ but its substitution with glycine generates a variant called Humanin G (HNG) that is 1000-fold more potent than its parent analog Humanin.^16^ HNG is known to protect against cell death by preventing mitochondrial dysfunction.^26^

Numerous studies highlight the cytoprotective role of HNG against Alzheimer’s disease and cancer,^26, 27^ but none have shed light on the role of HNG in protecting AMD mitochondria or AMD ARPE-19 transmitochondrial cybrid cells. Our cybrids were created by fusing human ARPE-19 *Rho0* (lacking mtDNA) cells with mitochondria from either AMD or age-matched normal subjects. Previously we have shown that despite all cell lines having identical nuclei, cybrids with AMD mitochondria express significantly different levels of complement RNA and proteins compared to normal cybrids.^28^ These changes can be attributed to variations in mtDNA, since all cybrids had similar nuclear content and were created using the same *Rho0* ARPE-19 cells. Furthermore, our previous studies have shown that mtDNA variants in cybrids can also affect the expression of inflammation, angiogenesis, and signaling genes.^29^

The present study demonstrates that cybrids containing AMD mitochondria have increased mtDNA fragmentation, impaired expression of mitochondrial replication/transcription genes, upregulation of pro-apoptotic genes and proteins, along with higher ROS levels and diminished cell viability compared to normal cybrids. Exposure to HNG reverses these events and protects the AMD mitochondria, promoting increased cellular longevity. Mechanistically, our results suggest that exogenous HNG acts via both intracellular (BAX) and extracellular (gp130) pathways and that retrograde signaling from AMD mitochondria to RPE cell nuclei contributes significantly to retinal cell death.

## Results

### AMD cybrids have damaged mitochondria

**Figure 1:**
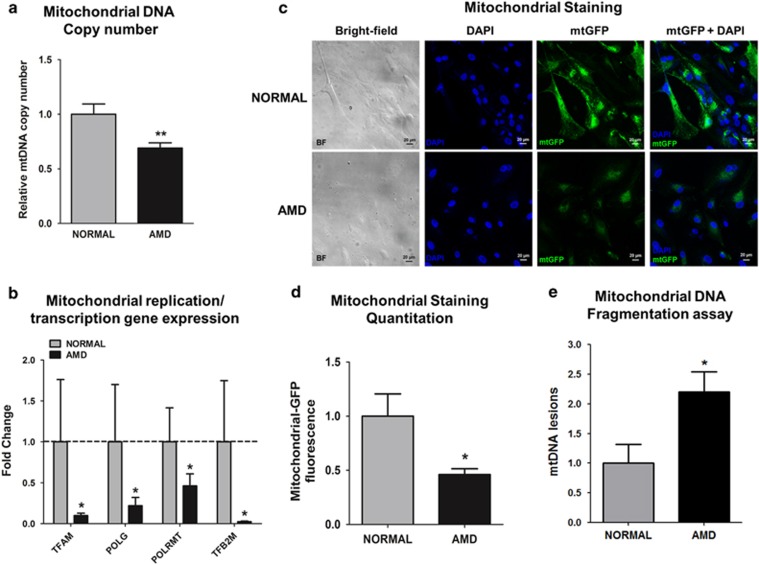
AMD cybrids have dysfunctional mitochondria. (**a**) AMD cybrids showed significantly reduced mitochondrial DNA (mtDNA) copy number compared to the age-matched normal cybrids (*n*=5, *P*=0.007). (**b**) AMD cybrids had lower expression levels of mitochondria replication and transcription genes. The AMD cybrids showed reduced expression of *TFAM* (90.1% decrease, *P*=0.04, *n*=5), *POLG* (78.1% decrease, *P*=0.03, *n*=5), *POLRMT* (53.8% decrease, *P*=0.03, *n*=5), and *TFB2M* (97.6% decrease, *P*=0.03, *n*=5) compared to normal cybrids (Supplementary Table S5). (**c**) Representative images of confocal microscopy showing diminished mtGFP staining throughout the cytoplasm in AMD cybrids compared to normal cybrids (Scale bar =20 *μ*m). (**d**) Quantitation of the 1C images showed that AMD cybrids had a 54% (*P*=0.04, *n*=4) decrease in mtGFP fluorescence compared to normal cybrids, suggesting lower numbers of AMD mitochondria. (**e**) AMD cybrids showed higher number of mtDNA lesions within the 503–2484 bps region compared to normal cybrids (*P*=0.04, *n*=5). Data are represented as mean±S.E.M., normalized to normal cybrids (assigned value of 1). The endpoint for all experiments was 72 h

To characterize mtDNA damage in AMD cybrids, the mtDNA copy numbers, expression levels of mt replication/transcription genes, mtGFP staining, and mtDNA fragmentation in normal and AMD cybrids were examined. Our results showed 31% reduction in mtDNA copy numbers in AMD cybrids (0.69±0.16 (mean±S.E.M.) arbitrary units (a.u.)) compared to normal cybrids (1±0.16 a.u., *P*=0.007) (Figure 1a). AMD cybrids showed decreased gene expression levels of *TFAM* (90.1%), *POLG* (78.1%), *POLRMT* (53.8%), and *TFB2M*(97.6%), compared to normal cybrids (*P*<0.05) (Figure 1b and Supplementary Table S5). When exposed to CellLight mt-GFP stain, AMD cybrids (0.46±0.05 a.u.) exhibited 54% decreased fluorescence compared to normal (1±0.2 a.u., *P*=0.04) (Figures 1c and d). Analyses of the 503-2484 bps mtDNA regions showed 120% higher numbers of mtDNA lesions in AMD cybrids (2.2±0.34 a.u.) compared to normal (1±0.32 a.u., *P*=0.04) (Figure 1e). Overall, these results suggest that mitochondria from AMD patients have significantly higher levels of mtDNA damage with impaired replication and transcription.

### AMD cybrids show decreased cellular viability and higher mt ROS production

**Figure 2:**
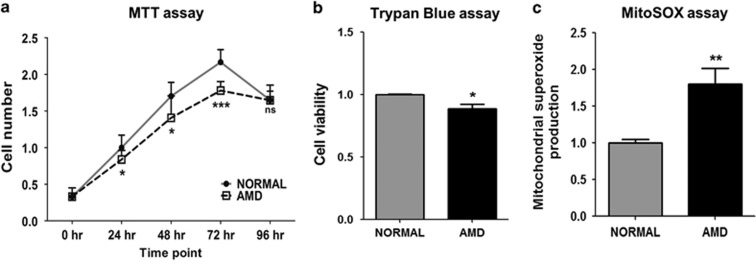
AMD cybrids show decreased cellular viability and higher mitochondrial (mt) ROS production. (**a**) The MTT assay demonstrated reduced numbers of viable cells in AMD cybrid cultures (24 h, 18% decline, *P*=0.03, *n*=4); (48 h, 17% decline, *P*=0.03, *n*=4); (72 h, 18% decline, *P*<0.001, *n*=4). At 96 h, the cell numbers in both normal and AMD cybrids declined and were similar to each other (*P*=0.98, *n*=4) (Supplementary Table S4). (**b**) When measured with Trypan blue dye exclusion assay, cell viability at 72 h declined significantly (11%, *P*=0.002, *n*=5) in AMD cybrids compared to normal cybrids (Supplementary Table S4). (**c**) MitoSOX assay at 72 h demonstrated higher mitochondrial ROS production in AMD cybrids compared to normal cybrids (80%, *P*=0.003, *n*=5) (Supplementary Table S4). Data are represented as mean±S.E.M., normalized to normal cybrids (assigned value of 1). The endpoint for all experiments was 72 h

Cell viability levels of normal and AMD cybrids were measured using both the MTT and Trypan blue dye exclusion assays. Using the MTT assay, AMD cybrids demonstrated significantly decreased cell viability at 24 h (18%), 48 h (17%), and 72 h (18%) compared to normal cybrids (*P*<0.05). At 96 h, normal and AMD cybrid values were similar to each other (*P*=0.98) (Figure 2a). The trypan blue assay results at 72 h showed 11% decrease in cell viability in AMD cybrids compared to normal (*P*=0.002, Figure 2b). The mitochondrial superoxide levels, as measured by the MitoSOX assay, were 80% higher in AMD cybrids compared to normal (*P*=0.003, Figure 2c) (Supplementary Table S4). These findings demonstrate increased oxidative stress in AMD cybrids and suggest that the AMD mitochondria cannot support cell growth at the rates seen in normal cybrids.

### AMD cybrids show differential expression of apoptosis, autophagy, ER stress, and antioxidant markers

**Figure 3:**
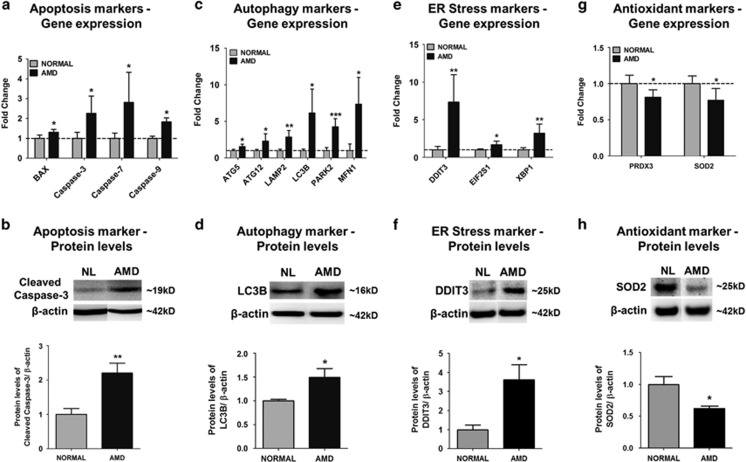
AMD cybrids showed differential expression levels of apoptosis, autophagy, ER stress, and antioxidant genes at 72 h. To examine the involvement of molecular/cellular pathways in mtDNA-mediated cell death, the gene expression profiles of apoptosis, autophagy, ER stress, and antioxidant markers were measured. (**a,c,e,g**) Using qRT-PCR analyses, the AMD cybrids showed upregulation of *Apoptosis genes* (**a**): *BAX* (30.8%, *P*=0.02, *n*=5), *Caspase-3* (125.7%, *P*=0.03, *n*=4), *Caspase-7* (181.3%, *P*=0.03, *n*=4–5), *Caspase-9* (82.8%, *P*<0.001, *n*=4–5); *Autophagy genes* (**c**): *ATG5* (54.4%, *P*=0.02, *n*=4), *ATG12* (130.5%, *P*=0.03, *n*=4), *LAMP2* (184.5%, *P*<0.001, *n*=4), *LC3B* (513.8%, *P*=0.01, *n*=4), *PARK2* (326.3%, *P*<0.001, *n*=4), *MFN1* (741.2%, *P*=0.02, *n*=5); and *ER stress genes* (**e**): *DDIT3* (633.9%, *P*=0.006, *n*=4), *eIF2α* (66.2%, *P*=0.03, *n*=4), *XBP1* (220.2%, *P*=0.005, *n*=4) compared to normal cybrids. AMD cybrids showed downregulation of *antioxidant genes* (**g**), *PRDX3* (18.8%, *P*=0.02, *n*=5) and *SOD2* (23.1%, *P*=0.04, *n*=3) in AMD cybrids (Supplementary Table S5). (**b**,**d**,**f**,**h)** Western blot analyses showed upregulated protein levels of Cleaved-Caspase-3 (120.4%, *P*=0.007, *n*=5; **b**), LC3B (49.1%, *P*=0.04, *n*=4; **d**), DDIT3 (261%, *P*=0.01, *n*=5; **f**), and downregulation of SOD2 (38%, *P*=0.01, *n*=5; **h**) in AMD cybrids (Supplementary Table S6). Data are represented as mean±S.E.M., normalized to normal cybrids (assigned value of 1). The endpoint for all experiments was 72 h

To determine if AMD mitochondria affect expression of cell death and cell stress markers, qRT-PCR and western blotting were performed. Compared to normal cybrids, the AMD cybrids showed *significant upregulation* of apoptosis genes (Figure 3a): *BAX* (30.8%), *Caspase-3* (125.7%) *Caspase-7* (181.3%), and *Caspase-9* (82.8%); *autophagy genes* (Figure 3c): *ATG5* (54.4%), *ATG12* (130.5%), *LAMP2* (184.5%), *LC3B* (513.8%), *PARK2* (326.3%), and *MFN1* (741.2%); and *ER stress genes* (Figure 3e): *DDIT3* (633.9%), *eIF2* (66.2%), and *XBP1* (220.2%). Furthermore, AMD cybrids showed downregulation of *antioxidant genes*, *PRDX3* (18.8%) and *SOD2* (23.1%) compared to normal (Figure 3g) (*P*<0.05) (Supplementary Table S5). Western blot analyses showed that the AMD cybrids had significantly higher protein levels of Cleaved Caspase-3 (120.4%, Figure 3b), LC3B (49.1%, Figure 3d), and DDIT3 (261%, Figure 3f); and 38% reduced SOD2 protein levels in AMD cybrids (Figure 3h) (*P*<0.05) (Supplementary Table S6).

Since all cybrids have identical nuclei, these results suggest that AMD mitochondria may mediate cell death/stress by upregulation of nuclear-encoded apoptosis, autophagy, and ER stress genes and downregulation of antioxidant genes.

### HNG reduces mtDNA-mediated apoptosis in AMD cybrids

**Figure 4:**
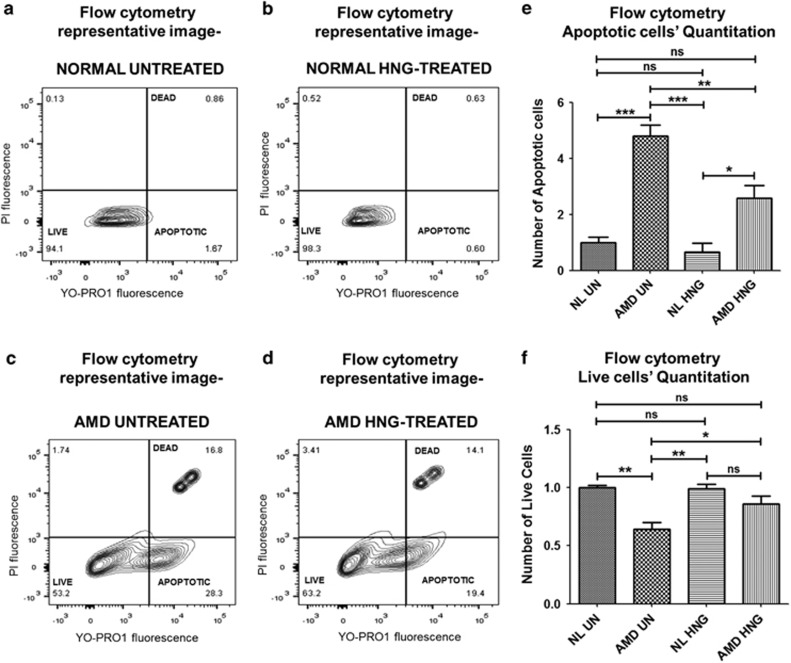
HNG reduces mitochondrial DNA-mediated apoptosis in cybrids. Flow cytometric analyses used YO-PRO1 and PI stains to identify apoptotic cells, live cells, and dead cells in AMD and normal cybrids treated with 3.2 *μ*M HNG for 72 h. Flow cytometry scatter plots of untreated normal (**a**) and untreated-AMD (**c**) cybrids along with HNG-treated normal (**b**) and HNG-treated AMD (**d**) cybrids are shown. The untreated-AMD cybrids had a 379% increase (*P*=0.001, *n*=3–4) in apoptotic cells (**e**) and a 36% decrease (*P*=0.0009, *n*=3–4) in live cells (**f**), compared to untreated normal cybrids. HNG treatment significantly decreased apoptotic cell numbers by 46.13% (*P*=0.02, *n*=3–4) in AMD cybrids compared to untreated AMD cybrids (**e**). No differences in the number of apoptotic cells and live cells were observed between untreated-normal and HNG-treated normal cybrids (Supplementary Table S7). Data are represented as mean±S.E.M., normalized to untreated-normal cybrids (assigned value of 1). The endpoint for all experiments was 72 h

To test the effects of exogenous HNG (3.2 *μ*M) on the numbers of live and apoptotic cells, cybrids were treated with YO-PRO-1 and propidium iodide stains, followed by flow cytometry analyses at 72 h. Figures 4a–d show representative profiles of untreated- and HNG-treated normal cybrids and AMD cybrids. Untreated-AMD cybrids had 379% increase in apoptotic cells (Figure 4e) and 36% decrease in live cells (Figure 4f) compared to untreated-normal cybrids (*P*<0.05). HNG drastically reduced the apoptotic cell number by 46.13% (Figure 4e) and increased the number of live cells by 34.37% (Figure 4f) in HNG-treated AMD cybrids compared to untreated-AMD cybrids (*P*<0.05) (Supplementary Table S7). No differences in either the apoptotic or live cell number were observed between untreated- and HNG-treated normal cybrids. Therefore, the AMD cybrids have higher levels of apoptotic and dead cells compared to cybrids with normal mitochondria. However, treatment with HNG can protect from apoptosis and elevate the levels of viable, healthy cells in AMD cybrids.

**Figure 5:**
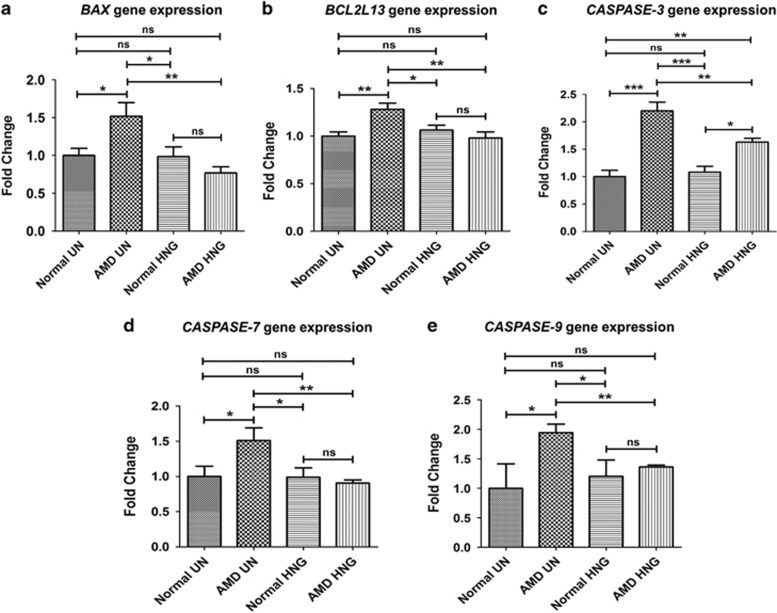
HNG downregulates expression of apoptosis genes in AMD cybrids as measured by qRT-PCR: In AMD cybrids, HNG significantly decreased the expression of apoptotic genes; *BAX* (49.4%, *P*=0.002, *n*=3–5; **a**), *BCL2L13* (23.69%, *P*=0.004, *n*=4; **b**), *Caspase-3* (25.85%, *P*=0.004, *n*=3; **c**), *Caspase-7* (39.83%, *P*=0.001, *n*=3–4; **d**), *Caspase-9* (29.94%, *P*=0.001, *n*=3–4; **e**), compared to untreated (UN)-AMD cybrids (Supplementary Table S8). Data are represented as mean±S.E.M., normalized to untreated-normal cybrids (assigned value of 1). The endpoint for all experiments was 72 h

In AMD cybrids, HNG treatment caused a significant decrease in the gene expression of apoptotic markers such as *BAX* (49.4%), *BCL2L13* (23.69%), *Caspase-3* (25.85%), *Caspase-7* (39.83%), and *Caspase-9* (29.9%) compared to untreated-AMD cybrids (*P*<0.05) (Figures 5a–e,Supplementary Table S8). No differences were found between untreated- *versus* HNG-treated normal cybrids. In summary, HNG reduced apoptosis in AMD cybrids, thereby highlighting its protective role in cybrids containing AMD mitochondria.

### HNG modulates the protein levels of BAX, gp130 receptor, and Phospho-JAK2 in AMD cybrids

**Figure 6:**
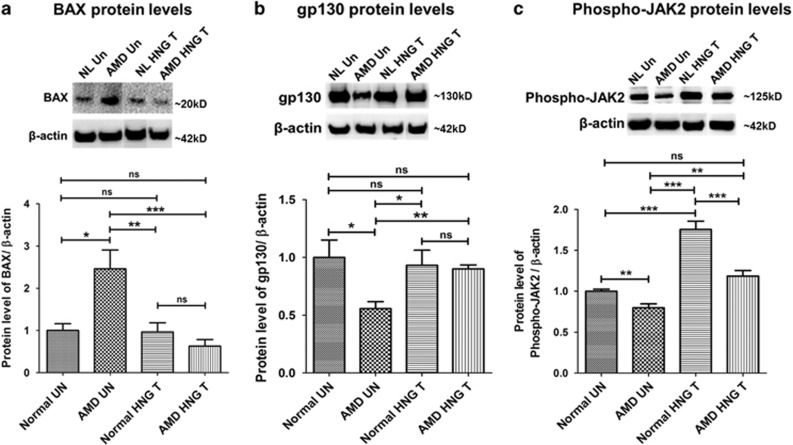
HNG effects via intracellular (BAX) and extracellular pathways (gp 130 and phospho-JAK2) in AMD and normal cybrids as shown by western blot analyses: BAX protein levels were 145.9% higher (*P*=0.04, *n*=3–5) in untreated (UN)-AMD cybrids than untreated (UN)-normal (NL) cybrids (**a**). HNG-treated AMD cybrids had lower BAX protein levels (74.42%, *P*=0.002, *n*=3–5) compared to untreated-AMD cybrids (**a**). Untreated-AMD cybrids showed 44.4% lower gp130 protein levels compared to untreated-normal cybrids (*P*=0.03, *n*=3–4; **b**). HNG-treated AMD cybrids had 61.87% increased levels of gp130 protein compared to untreated-AMD cybrids (*P*=0.007, *n*=3–4; **b**) Phospho-JAK2 protein levels were 20.3% lower in untreated-AMD cybrids compared to untreated-normal cybrids (*P*=0.009, *n*=4; **c**). HNG-treated AMD cybrids showed 48.05% higher phospho-JAK2 protein levels than untreated-AMD cybrids (*P*=0.003, *n*=4; **c**) (Supplementary Table S9). Data are represented as mean±S.E.M., normalized to untreated-normal cybrids (assigned value of 1). The endpoint for all experiments was 72 h

To assess mechanisms by which HNG protects AMD cybrids, the protein levels of BAX (acts intracellularly), gp130 (a transmembrane glycoprotein and cell surface receptor), and phosphorylated (Phospho)-JAK2 (a signaling molecule and a marker of activated JAK) were compared. As shown in Figure 6a, BAX was significantly elevated (145.9%, *P*=0.04) in untreated AMD cybrids compared to untreated normal cybrids. In AMD cybrids, HNG decreased BAX protein levels by 74.42% compared to untreated-AMD cybrids (*P*=0.002) (Supplementary Table S9).

Untreated-AMD cybrids showed 44.4% decrease in gp130 (*P*=0.03, Figure 6b) and 20.25% decrease in Phospho-JAK2 (*P*=0.009, Figure 6c), compared to untreated-normal cybrids. HNG treatment increased gp130 by 61.87% (*P*=0.007, Figure 6b) and Phospho-JAK2 by 48.05% (*P*=0.003, Figure 6c) in AMD cybrids compared to untreated-AMD cybrids. The gp130 protein levels were unchanged (Figure 6b) and the Phospho-JAK2 protein levels were 32.57% lower (*P*=0.003, Figure 6c) in HNG-treated AMD cybrids compared to HNG-treated normal cybrids (Supplementary Table S9).

These results demonstrate that: (1) AMD cybrids have altered BAX and gp130 protein levels, which can be restored to normal levels with HNG treatment, and (2) AMD cybrids have reduced Phospho-JAK levels, and HNG treatment activates JAK, thereby signaling the transcription of downstream protective genes and mediating cytoprotection.

### HNG reduces amyloid *β*-induced and mtDNA-mediated cell stress in AMD cybrids

To determine if HNG protected against amyloid *β*-induced cell stress, the normal and AMD cybrids were treated with either HNG alone, amyloid-*β*_1–42_ (active form) or amyloid-*β*_42–1_ (inactive scrambled control) peptides alone or a combination of HNG plus amyloid-*β*. Cell viability was measured using the MTT assay.

**Figure 7:**
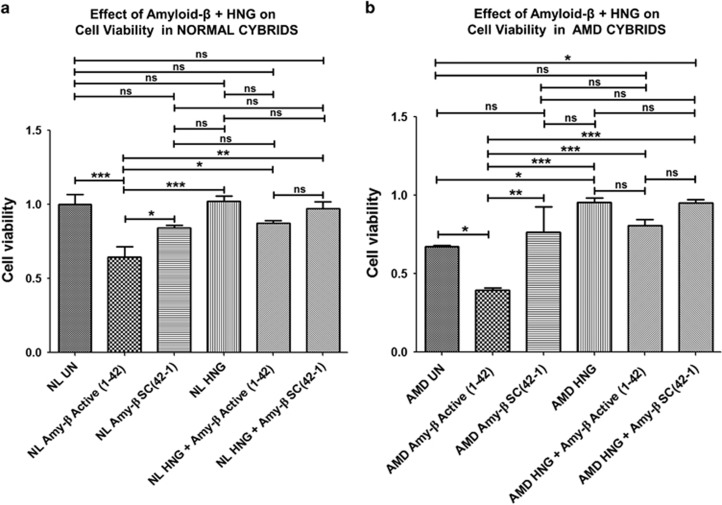
HNG reduces amyloid *β*-induced and mtDNA-mediated cell stress: Protective effects of pre-treatment with HNG against amyloid-*β* cytotoxicity were measured using the MTT assay. *Amy-β* *Active (1-42)* represents Amyloid-*β*_1–42_ which is the active form; *Amy-β SC(42-1)* represents amyloid-*β*_42-1_ which is the scrambled, inactive form that serves as control. (**a**) Compared to untreated (UN)-normal (NL) cybrids, *Amy-β* *Active (1-42)*-treated NL cybrids showed a 35% (*P*<0.001, *n*=4) reduction in cell viability. No differences in cell viability were observed between untreated-NL cybrids and *Amy-β SC(42-1)*-treated NL or HNG-treated NL. Cell viability was significantly higher (31.25%, *P*<0.05, *n*=4) for *Amy-β SC(42-1)*-treated NL cybrids *versus Amy-β* *Active (1-42)*-treated NL cybrids. HNG increased cell viability by 35.94% (*P*<0.05, *n*=4) in *Amy-β* *Active (1-42)*-treated NL cybrids compared to the NL cybrids treated with *Amy-β* *Active (1-42)*-treated alone (Supplementary Table S10A). (**b**) Treatment of AMD cybrids with *Amy-β* *Active (1-42)* alone reduced cell viability by 41.8% (*P*<0.05, *n*=3–4) compared to untreated-AMD cybrids. No difference in cell viability was observed between untreated-AMD cybrids and *Amy-β SC(42-1)*-treated AMD cybrids. Significant reduction in cell viability was observed in *Amy-β* *Active (1-42)*-treated AMD cybrids (94.9%, *P*<0.01, *n*=3–4) compared to the *Amy-β SC(42-1)*-treated AMD cybrids. AMD cybrids treated with HNG alone had a 42.4% increase in cell viability compared to untreated-AMD cybrids (*P*<0.05, *n*=3–4). Pre-treatment with HNG increased cell viability by 107.7% in *Amy-β* *Active (1-42)*-treated AMD cybrids (*P*<0.001, *n*=3–4) compared to the AMD cybrids treated with active *Amy-β* *Active (1-42)* alone (Supplementary Table S10B). Data are represented as mean±S.E.M., normalized to untreated-normal cybrids (assigned value of 1). The endpoint for all experiments was 72 h

Figure 7a demonstrates that HNG can protect normal cybrids against the cytotoxic effects of amyloid-*β*. Normal cybrids treated with amyloid-*β*_1–42_ (active form, bar 2) showed reduced cell viability compared to untreated-normal (35%, *P*<0.001, bar 1) and amyloid-*β*_42-1_-treated (scrambled form) normal cybrids (31.25%, *P*<0.05, bar 3). Untreated-normal cybrids (bar 1) had similar levels of cell viability as amyloid-*β*_42-1_-treated (scrambled form, bar 3) and HNG-treated normal cybrids (bar 4). HNG increased cell viability by 35.94% in amyloid-*β*_1–42_-treated normal cybrids (bar 5) compared to the amyloid-*β*_1–42_-treated normal group (*P*<0.05, bar 2) (Supplementary Table 10 A).

HNG can also protect AMD cybrids against the cytotoxic effects of amyloid-*β* (Figure 7b). The amyloid-*β*_1–42_-treated AMD cybrids (active form, bar 2) showed 41.8% reduced cell viability compared to untreated-AMD (*P*<0.05, bar 1) and 94.9% reduction compared to amyloid-*β*_42-1_-treated (scrambled form) AMD cybrids (*P*<0.01, bar 3). Untreated-AMD cybrids (bar 1) had similar levels of cell viability as amyloid-*β*_42-1_-treated (scrambled form, bar 3). HNG-treated AMD (bar 4) cybrids had 42.4% increased cell viability compared to untreated-AMD cybrids (bar 1, *P*<0.05). AMD cybrids treated with HNG plus amyloid-*β*_1–42_ (bar 5) showed 107.7% increased cell viability compared to amyloid-*β*_1–42_-treated-AMD cybrids (*P*<0.001, bar 2) (Supplementary Table 10 B). Our findings demonstrate that HNG was protective in AMD cybrids treated with amyloid-*β*_1–42_ (active form).

### HNG prevents loss of AMD mitochondria

**Figure 8:**
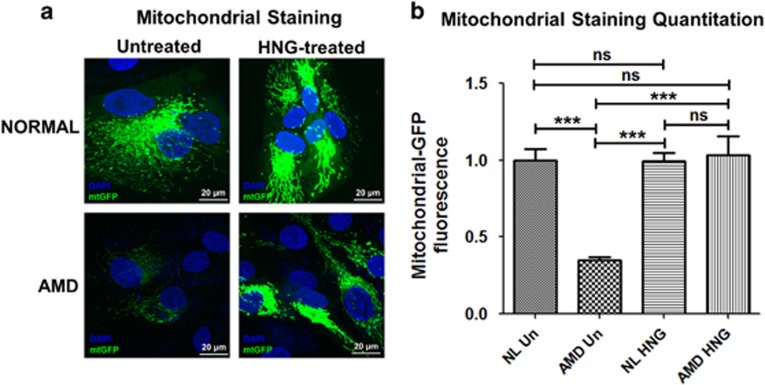
HNG prevents loss of AMD mitochondria: (**a**) Confocal images of cybrids treated with mtGFP stain demonstrated increased mtGFP fluorescence in HNG-treated AMD cybrids. (**b**) In AMD cybrids, quantification graphs show that HNG increases mtGFP fluorescence by 194.3% (*P*<0.001, *n*=5) compared to AMD cybrids. The HNG-treated normal cybrids and untreated-normal cybrids showed similar levels of mtGFP staining (Supplementary Table S11). Scale bar=20 *μ*m. Data are represented as mean±S.E.M., normalized to untreated-normal cybrids (assigned value of 1). The endpoint for all experiments was 72 h

Confocal microscopy of AMD cybrids stained with CellLight mtGFP fluorescent probe for 48 h showed higher levels of mitochondria in HNG-treated AMD cybrids (Figures 8a and b). Significant increase in fluorescent intensity (194.3%, *P*<0.001) was observed in HNG-treated AMD cybrids compared to untreated-AMD cybrids. No difference was observed between untreated-normal and HNG-treated normal cybrids (Supplementary Table S11). This finding indicates that HNG can increase mitochondria-targeted staining in AMD cybrids.

## Discussion

In the current study, we used the transmitochondrial ARPE-19 cybrid model to identify and characterize the effects of AMD mitochondria on human retinal cells. Through a combination of cell-based and molecular biology assays, we found that cellular/mitochondrial damage occurred at multiple levels in AMD cybrids, and that HNG protected AMD cybrids from mtDNA mediated and amyloid-*β*-induced cellular stress.

Previous studies have shown that mtDNA-deficient (*Rho0*) ARPE-19 cells had diminished mitochondrial membrane potential, altered expression of genes related to lipid transport, inflammation, drusen deposits and various extracellular matrix materials compared to wild-type cells.^30^ This indicates that the mitochondria play a role in those particular pathways. The present study used these same *Rho0* ARPE-19 cells as the host cell to create the cybrids using mitochondria from either AMD or age-matched normal subjects. While the preparation of *Rho0* cells may affect the nuclear (n) DNA, the cell has efficient, rapid nDNA repair mechanisms which the mtDNA lacks. With the introduction of the AMD or normal mitochondria into the uniform *Rho0* ARPE-19 cell line, one can assume that alterations in molecular or biochemical behavior seen in the cybrids are due to the variations of the mtDNA rather than nDNA. To test consistency, we have created different cybrid batches using mitochondria from the same subject and the gene expression patterns, cell viability and membrane potentials were similar in the cell lines for the individuals. Therefore, our cybrid model represents a ‘personalized’ model that is reliable and uniform for each subject and can be used to screen mitochondria-targeting drugs. Furthermore, the additional significant changes in cell-death related genes observed in AMD cybrids could be attributed to unhealthy AMD mitochondria.

AMD pathology is characterized by RPE cell death, which precedes geographic atrophy and leads to eventual deterioration of the overlying photoreceptors.^31^ In addition, RPE cell death induced by various stressors such as hydrogen peroxide (tBHP), BMP4, and hypoxia has been demonstrated in *in vitro* AMD models,^32, 33, 34^ and in several retinal degeneration animal models.^35, 36^ Consistent with those findings, our results with Trypan blue and MTT cell viability assays demonstrated substantial increase in AMD cybrid cell death at 24h, 48h, and 72 h time points. Since all cybrids contained similar nuclei and differed only in mtDNA, these results indicated that mitochondria from AMD patients were dysfunctional and mediated cell death. RPE cells are highly metabolically active and contain large numbers of mitochondria, which are the primary source of endogenous ROS.^37^ Our results revealed that AMD cybrids have: (1) elevated levels of mitochondrial superoxides, the predominant ROS found in the mitochondria, and (2) reduced expression of antioxidant genes, *PRDX3* and *SOD2*. Together these data represent an imbalance between mitochondrial ROS production and antioxidant enzyme levels, which would lead to significantly higher oxidative stress within AMD cybrids compared to the controls. Our results are consistent with previous studies showing that damaged mitochondria and ageing lead to increased ROS and oxidative damage and reduced antioxidant capacity both in *in vitro* and *in vivo* models of retinal diseases.^38, 39^

Analyses of transcript profiles indicated that cybrids with mitochondria from AMD subjects have higher levels of apoptosis, autophagy and ER stress compared to cells possessing normal mitochondria, suggesting that the mechanism of cell death mediated by AMD mitochondria is multifactorial. Our recent study also showed AMD mitochondria altered the expression of complement pathway markers in AMD cybrids.^28^ It seems clear that damaged, unhealthy AMD mitochondria transmit retrograde signals to mediate expression patterns of nuclear-encoded genes in cybrids but the exact mechanisms are not yet known. Possible pathways include changes in epigenetics mediated by mtDNA variants,^29, 40, 41^ and mtDNA fragments that upregulate inflammatory genes’ expression, via the STING (Stimulator of Interferon Genes) pathway.^42^ To cope with perturbations in cellular homeostasis within retinal cells, apoptosis, unfolded protein response due to ER stress, and autophagy are triggered.^43, 44, 45^ Additionally, apoptosis- and ER stress-mediated cell death of RPE has been documented in various retinal layers in retinal degenerative diseases, including AMD.^46, 47^ However, to our knowledge, ours is the first study demonstrating that platelet-derived mitochondria from AMD patients can significantly alter cell survival, reflecting similar events that occur in the AMD retinas.

Feher *et al.* used morphometric analyses of transmission electron micrographs to show that mitochondria of human primary RPE cells from AMD eyes were decreased in number and size, had disrupted cristae, disrupted mitochondrial membranes, and extensive reduction in matrix density compared to the controls.^48^ In our study, multiple techniques were used to demonstrate that AMD mitochondria are damaged compared to age-matched normal. AMD cybrids exhibited a significant decrease in mitochondrial GFP fluorescence, lower mtDNA copy numbers and increased mtDNA fragmentation compared to normal cybrids. Furthermore, AMD cybrids had downregulation of *TFAM*, *POLG*, *POLRMT*, and *TFB2M* genes, suggesting impaired mitochondrial transcription/replication. Cytoprotective MDPs, such as Humanin and SHLPs, are encoded in the mitochondrial genome at the *16S* rRNA gene.^15^ Real-time PCR with primers spanning 503–2484 bps mtDNA regions showed increased numbers of mtDNA lesions in this region of the AMD mitochondrial genome. Our results are consistent with Ferrington *et al.*’s that demonstrated elevated levels of mtDNA lesions in specific regions, including MDP-coding regions of the mitochondrial genome, in AMD primary human RPE cells.^49^ Other studies have also established a link between human RPE mtDNA damage and pathologic ageing in AMD.^7^ It may be that mtDNA fragmentation disrupts the integrity of mitochondria and the threshold levels of MDPs essential for maintaining mitochondrial/cellular health and longevity.

In order to rescue and restore homeostasis to cells, we hypothesized that cellular and mitochondrial damage observed in AMD cybrids could be prevented by treatment with HNG, a more potent variant of Humanin. To that end, AMD and normal cybrids were treated with 3.2 *μ*M HNG, co-stained with YO-PRO1/Propidium iodide and the apoptotic cell percentage measured using flow cytometry. As predicted, treatment of AMD cybrids with HNG led to a drastic decrease in numbers of apoptotic cells, confirming that HNG protected AMD cybrids against mitochondria-mediated cell death. These results are similar to Hinton *et al.*’s study showing Humanin was capable of protecting primary human RPE cells against oxidative stress-induced and ER stress-induced cell death,^24^ using tert-Butyl hydroperoxide and tunicamycin, respectively.

Having confirmed the cytoprotective role of HNG on quiescent cybrids containing AMD mitochondria, we next determined the effects of HNG on cybrids stressed by exposure to amyloid-*β* peptides. Significant cytotoxicity was demonstrated in normal and AMD cybrids exposed to the active amyloid-*β*_1-42_ peptide. However, pretreatment with HNG protected both AMD and normal cybrids from amyloid-*β*_1-42_-induced toxicity. These findings are significant because amyloid-*β* is a key constituent of drusen, which are sub-RPE deposits composed primarily of lipid and proteins.^50^ Previous studies have demonstrated that cleavage of APP (amyloid precursor protein) by proteases produces the long forms of amyloid-*β* (i.e.,amyloid-*β*_42_) which are fibrillogenic, plaque-forming and associated with neurodegenerative disease states, including Alzheimer’s disease.^51, 52^ Our findings agree with studies reporting the cytoprotective roles of Humanin and HNG against amyloid-*β*-induced cell death in numerous aging-related diseases.^53^ For instance, Hashimoto *et al.* demonstrated that Humanin inhibits neuronal cell death induced by amyloid-*β*, mutant APP, and presenilin.^54, 55^ Zhang *et al.* showed that administration of HNG attenuates neuro-inflammation, cognitive deficits, and amyloid-*β* burden in a transgenic mouse model of Alzheimer’s disease.^56^ Humanin and/or HNG have also been shown to offer protection in cardiovascular, metabolic, inflammation, and cancer disease models.^20, 57, 58^

One mechanism by which HNG protects AMD cybrid cells from death may be through stabilization of mitochondria and prevention of mitochondrial death. HNG-treated AMD cybrids had 197.1% increase in mtGFP fluorescence compared to untreated-AMD cybrids, demonstrating that the AMD mitochondria can be rescued by HNG pretreatment. This finding agrees with a previous study showing Humanin protects mitochondria in primary RPE cells.^24^

Next, we investigated the mechanisms by which HNG exerts its cytoprotective effects. Humanin affects cells via both intracellular and extracellular mechanisms.^13^ Humanin can bind BAX protein, which prevents the coupling of tBid with BAX, and the release of cytochrome *c*, thereby inhibiting apoptosis.^59^ In untreated-AMD cybrids, the BAX protein levels were significantly elevated by 146% but declined drastically by 74% after HNG treatment. These findings indicate that HNG exerts its cytoprotective action via the intracellular mechanism involving BAX protein. With respect to the extracellular mechanism, Humanin is known to bind to a trimeric CNTFR/gp130/WSX-1 receptor complex, inducing activation of JAK-STAT pathway and transcription of downstream genes.^60^ In the current study, untreated-AMD cybrids had significantly reduced gp130 protein compared to untreated-normal. However, addition of HNG led to 61.87% increase in gp130 protein levels compared to untreated-AMD cybrids, thus restoring levels to that seen in normal cybrids. This suggests that AMD cybrids may have either a low abundance of the trimeric receptor or perhaps a faulty receptor complex which does not allow Humanin to bind and exert its cytoprotective effects. We speculate that gp130 and the trimeric receptor complex is associated with HNG-induced rescue of AMD cybrids. Furthermore, the initially low levels of phospho-JAK2 protein were restored with HNG in normal and AMD cybrids, suggesting that restoring gp130 levels may mediate activation of JAK and promotes cytoprotective effects.

To conclude, this novel study demonstrates that mitochondria from AMD patients are severely damaged, and highlights the protective role of HNG against mitochondria-mediated and amyloid-*β*-induced cell death in AMD transmitochondrial ARPE-19 cybrids. Our findings support the hypothesis that HNG is a significant cell survival factor and should be considered as a potential therapy for the treatment of dry AMD. These results also highlight the potential beneficial properties of Humanin and other MDPs that have been characterized in non-ocular disease models but have not yet been investigated in AMD and other age-related eye diseases.

## Materials and methods

### Human subjects

Research involving human participants was approved by the Institutional review board of the University of California, Irvine (#2003-3131). Written informed consent was obtained from all subjects (Supplementary Table S1) and clinical investigations were conducted according to the tenets of Declaration of Helsinki for research involving human subjects. We found no significant difference (*P*=0.07) in ages of normal (72.60±1.7 years) and AMD (77.60±1.6 years) subjects (*N*=5).

### Transmitochondrial cybrids

Transmitochondrial cybrids were created by fusing mitochondrial DNA-deficient APRE-19 (*Rho0*) cell line with platelets isolated from either AMD patients or age-matched normal subjects. Peripheral blood (10 ml) was collected via venipuncture in tubes containing 10 mM EDTA. Blood platelets were isolated by a series of centrifugation steps, in tubes containing 3.2% sodium citrate, and final pellets were suspended in Tris buffered saline. To create the ARPE-19 *Rho0* cells, ARPE-19 cells were serially passaged into medium containing 50 ng/ml ethidium bromide.^30^ Cybrids were created by polyethylene glycol fusion of platelets with *Rho0* ARPE-19 cells in medium containing uridine. After growing the cybrid cells in uridine-containing medium for 2 weeks, the medium was replaced with regular culture medium. Mitochondrial DNA haplogroups profiles of each cybrid were confirmed using PCR, restriction enzyme digestion, and sequencing. The SNPs defining the H haplogroup were T7028C and G73A. All transmitochondrial cybrids were grown in DMEM/Ham’s F12 1:1 (Invitrogen-Gibco, Gaithersburg, MD, USA) cell culture medium containing 24 mM sodium bicarbonate, 10% dialyzed fetal bovine serum, and 1.0 mM sodium pyruvate. Passage 5 cybrids were used for all experiments. Age-matched normal cybrids served as controls.

### Treatment with Humanin G (HNG)

Lyophilized HNG (Anaspec, Fremont, CA, USA) was reconstituted in water to obtain a stock solution that was subsequently dissolved in culture media to obtain a 3.2 *μ*M HNG working solution. In this study, all cybrids were treated with 3.2 *μ*M HNG. At the beginning of this study we tested a wide range of concentrations (based on the published Humanin literature) ranging from 0.8 to 10 *μ*M to determine a low optimum working concentration of HNG in our ARPE-19 cybrid cells. The HNG concentrations of 0.8 and 1.6 *μ*M did not show any protective effects. Our preliminary studies demonstrated that 3.2 *μ*M was the lowest concentration that showed significant protective effects in the cybrid cells. Therefore, we used 3.2 *μ*M as our final working concentration for all experiments with HNG.

### MTT assay

The MTT assay is a colorimetric test that utilizes the phenomenon of reduction of tetrazolium salts to measure cell viability. Healthy actively metabolizing cells contain NAD(P)H-dependent cellular oxidoreductase enzymes that reflect the number of viable cells present. These enzymes reduce the water-soluble yellow tetrazolium dye MTT [3-(4,5-dimethylthiazol-2-yl)-2,5-diphenyltetrazolium bromide] to its insoluble formazan, which has a purple color. The formazan crystals are then solubilized by adding dimethyl sulfoxide (DMSO) and the colorimetric signal, which is proportional to the number of viable cells, is determined by measurement of optical density at 570 nm. MTT assays were performed in the dark since the MTT reagent is sensitive to light.

AMD and normal cybrids (*n*=4-5) plated in 96-well tissue culture plates were treated with MTT solution (Cat.# 30006, Biotium, Fremont, CA, USA) at 24, 48, 72, and 96 h time points. MTT-treated cells were incubated at 37 °C for 1 h, followed by addition of DMSO. Absorbance was measured on a spectrophotometer at 570 nm and background absorbance measured at 630 nm. Background absorbance was subtracted from signal absorbance to obtain normalized absorbance values. The colorimetric signal obtained was proportional to the cell number.

### Trypan Blue dye exclusion assay

Trypan blue is a diazo dye that selectively stains dead cells blue. Trypan blue dye exclusion test is based on the principle that live cells that have intact cell membranes are impervious to Trypan blue dye; therefore viable cells are excluded from staining blue. Since in our study, cell growth of cybrids peaked at 72 h, AMD and normal cybrids (*n*=5) were plated in six-well plates, trypsinized at 72 h, and transferred to a counting vial. Cell count in each sample was determined using a cell counter (ViCell, Beckman Coulter, Brea, CA, USA) that uses Trypan blue dye to determine the number of viable cells. A total of 50 images were captured and averaged per sample.

### MitoSOX assay

The MitoSOX Red reagent is a live-cell permeant fluorogenic dye that allows highly selective detection of mitochondrial superoxides in live cells. Once the MitoSOX Red reagent is oxidized by superoxides, it exhibits red fluorescence that can be quantified. AMD and normal cybrids (*n*=5) were plated in 24-well tissue culture plates for 72 h. The 5 mM MitoSOX reagent (Cat.# M36008, Invitrogen, Grand Island, NY, USA) stock solution was diluted with HBSS (Hank’s balanced salt solution) buffer to make a 5 *μ*M MitoSOX reagent working solution. Cells were treated with 5 *μ*M MitoSOX reagent and incubated for 10 min at 37 °C. Cells were then washed with HBSS buffer, and fluorescence was measured at excitation/emission maxima of 510/580 nm.

### Confocal microscopy

To estimate the number of mitochondria in normal and AMD cybrid cells, CellLight mtGFP staining was performed. The CellLight Mitochondrial GFP stain (Cat. # C10582, Thermo Fisher Scientific, Waltham, MA, USA) is a baculovirus fusion construct having a mammalian promoter and a leader sequence of E1 alpha pyruvate dehydrogenase fused to GFP. Therefore, it specifically targets mitochondria and mtGFP fluorescence can be quantified. Normal and AMD cybrids (*n*=5) were plated in four-well tissue culture chamber slides for 72 h. At 48 h, CellLight stain was added to the medium at a concentration based on the cell density at the end of incubation period. The cells in chamber slides were incubated overnight at 37 °C. The cells were washed with 1 × TBS (Tris-buffered saline), fixed in paraformaldehyde, washed again with 1 × TBS, mounted in DAPI, coverslipped, and stored at 4 °C. Confocal z-stack images were captured using the LSM-700 Confocal microscope (Zeiss, Thornwood, NY, USA). All confocal images were quantified using the inbuilt ZEN 2 lite software.

### Mitochondrial copy number

Normal and AMD cybrids (*n*=5) were grown in six-well plates, and total DNA was isolated at 72 h after plating. Quantitative real-time PCR was performed using TaqMan gene expression assays for 18S and MT-ND2 (Cat. # 4331182, Thermo Fisher Scientific) genes and TaqMan gene expression master mix (Cat. # 4369016, Thermo Fisher Scientific). Relative mtDNA copy numbers were determined using delta Cts. All samples were run in triplicates.

### Mitochondrial DNA fragmentation assay

To examine mitochondrial lesions in cybrid DNA, real-time PCR was performed with total DNA isolated at 72 h from normal (*n*=5) and AMD cybrids (*n*=5) using primers covering the MT-12S and MT-16S region of the mtDNA (Forward primer: 5′-ATCCTACCCAGCACACAC-3′ Reverse primer: 5′-GATTTGCCGAGTTCCTTTTACT)-3′, and the Accuprime TaqMan DNA Polymerase System (Cat. # 12339016, Thermo Fisher Scientific). PCR products were run on 1% agarose gels and imaged under UV light. Number of mtDNA lesions were counted and compared between normal and AMD groups.

### Quantitative real-time PCR (qRT-PCR)

RNA extraction and cDNA synthesis: Normal and AMD cybrid cells (*n*=5) were grown in six-well plates, and RNA was extracted at 72 h using the RNeasy Mini Kit (Qiagen, Valencia, CA, USA) as per the manufacturer’s protocol. The extracted RNA was quantified using NanoDrop 1000 Spectrophotometer (Thermoscientific, Waltham, MA, USA). RNA (100 ng/*μ*l) was reverse transcribed into cDNA using Superscript VILO Master Mix (Cat. # 11755-050, Invitrogen). Diluted cDNA was stored at –20 °C until used.

### qRT-PCR

qRT-PCR was performed using StepOnePlus Real-Time PCR system (Applied Biosystems, Carlsbad, CA, USA) to study the expression of apoptosis, autophagy, ER stress, and antioxidant genes. Each sample was run in triplicate. The QuantiTect Primer Assays (Qiagen) (Supplementary Table S2) and Power SYBR Green PCR Master Mix (Cat. # 4367659, Life Technologies, Grand Island, NY, USA) were used. Specific housekeeper genes used are listed in Supplementary Table S2. qRT-PCR data were analyzed using ΔΔCt method. ΔCt was the difference between the Cts (threshold cycles) of the target gene and Cts of the housekeeper gene (reference gene). ΔΔCt was calculated by subtracting ΔCt of the AMD group from ΔCt of the normal group. Fold change was calculated using the following formula: Fold change =2^ΔΔCt^.

### Western blotting analyses

#### Protein extraction

Normal and AMD cybrid cells (*n*=3–5) were plated in six-well plates for 72 h. Cells were lysed using RIPA buffer (Cat. # 89900, Life Technologies), supernatants transferred to a new microfuge tube and concentrations of proteins were measured using Bio-Rad Dc protein assay system (Bio-Rad Laboratories, Richmond, CA, USA) according to the manufacturer's instructions.

#### Immunoblotting

Equal concentrations of total protein samples were loaded into the wells of 4–12% Bolt mini gels (Life Technologies) followed by SDS-PAGE electrophoresis. The gels were then transferred onto PVDF membranes. Following transfer, the membranes were blocked in 5% milk for 1 h, then incubated overnight at 4^o^C in primary antibodies (Supplementary Table S3). Blots were then washed with 1 × TBST (Tris Buffered Saline-Tween20) and incubated with the respective secondary antibodies for 1 h at room temperature. All primary and secondary antibodies were diluted in 1 × TBST. Following secondary antibody incubation, the blots were washed with 1 × TBST. Protein bands were detected using Clarity Western ECL Blotting Substrate (Cat. #1705060, Bio-Rad). *β*-actin antibody was used as a housekeeper protein control. Protein bands were visualized using Versadoc imager (Bio-Rad), and quantified using ImageJ software (NIH Image).

### Flow cytometry

Normal and AMD cybrids (*n*=4–5) were stained with YOPRO-1/propidium iodide (PI) (Cat. # V13243, Invitrogen), and incubated on ice for 20–30 min. Following incubation, stained cells were analyzed by flow cytometry, using 488 nm excitation with green fluorescence emission for YOPRO-1 (i.e., 530/30 bandpass) and red fluorescence emission for PI (i.e., 610/20 bandpass), gating on cells to exclude debris. Standard compensation analysis using single-color stained cells was performed. The YO-PRO-1 dye (green fluorescent) can enter apoptotic cells, whereas PI (red fluorescent dye) cannot. Therefore, YO-PRO-1 dye and PI together provide a sensitive indicator for apoptosis. Live cells show a low level of green fluorescence, apoptotic cells show an incrementally higher level of green fluorescence, and dead cells show both red and green fluorescence.

### Treatment with amyloid-*β* peptides

Lyophilized amyloid-*β*_1–42_ (active form) (Cat. # AS-20276, Anaspec) and amyloid-*β*_42–1_ (inactive scrambled control) peptides (Cat. # 27276, Anaspec) were reconstituted in 1% ammonium hydroxide to prepare a stock solution, that was subsequently dissolved in 1 × PBS (phosphate-buffered saline) to obtain a 20 *μ*M working solution. All cybrids (*n*=4–5) were treated for 24 h with 20 *μ*M amyloid-*β* peptides in this study.

### Statistical analysis

Results between groups were analyzed for differences using the unpaired Student’s *t*-test or one-way ANOVA followed by Tukey–Kramer *post-hoc* test (GraphPad Prism 5.0; GraphPad Software, CA, USA). Statistical significance was determined at *P*-values <0.05.
