# Supplementary material for: Humanin G (HNG) protects age-related macular degeneration (AMD) transmitochondrial ARPE-19 cybrids from mitochondrial and cellular damage
Source: Cell Death Dis. 2017 Jul 20;8(7):e2951–. doi: 10.1038/cddis.2017.348 (PMC5550888; doi:10.1038/cddis.2017.348)
Supplement: Supplementary Tables [file cddis2017348x1.docx]

**Supplementary Information:**

**Table S1. List of Patients and Controls used to create normal and AMD cybrids**

**
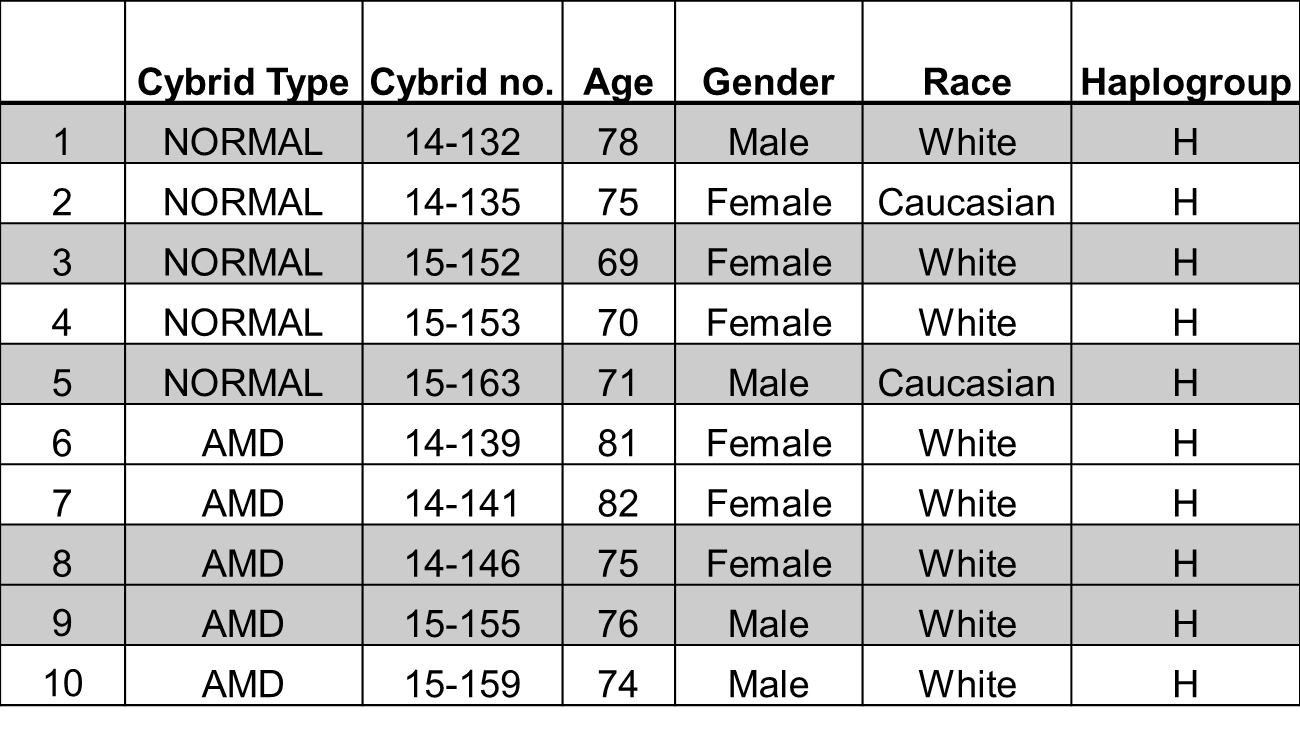
**

**Table S2. List of Primers**

**
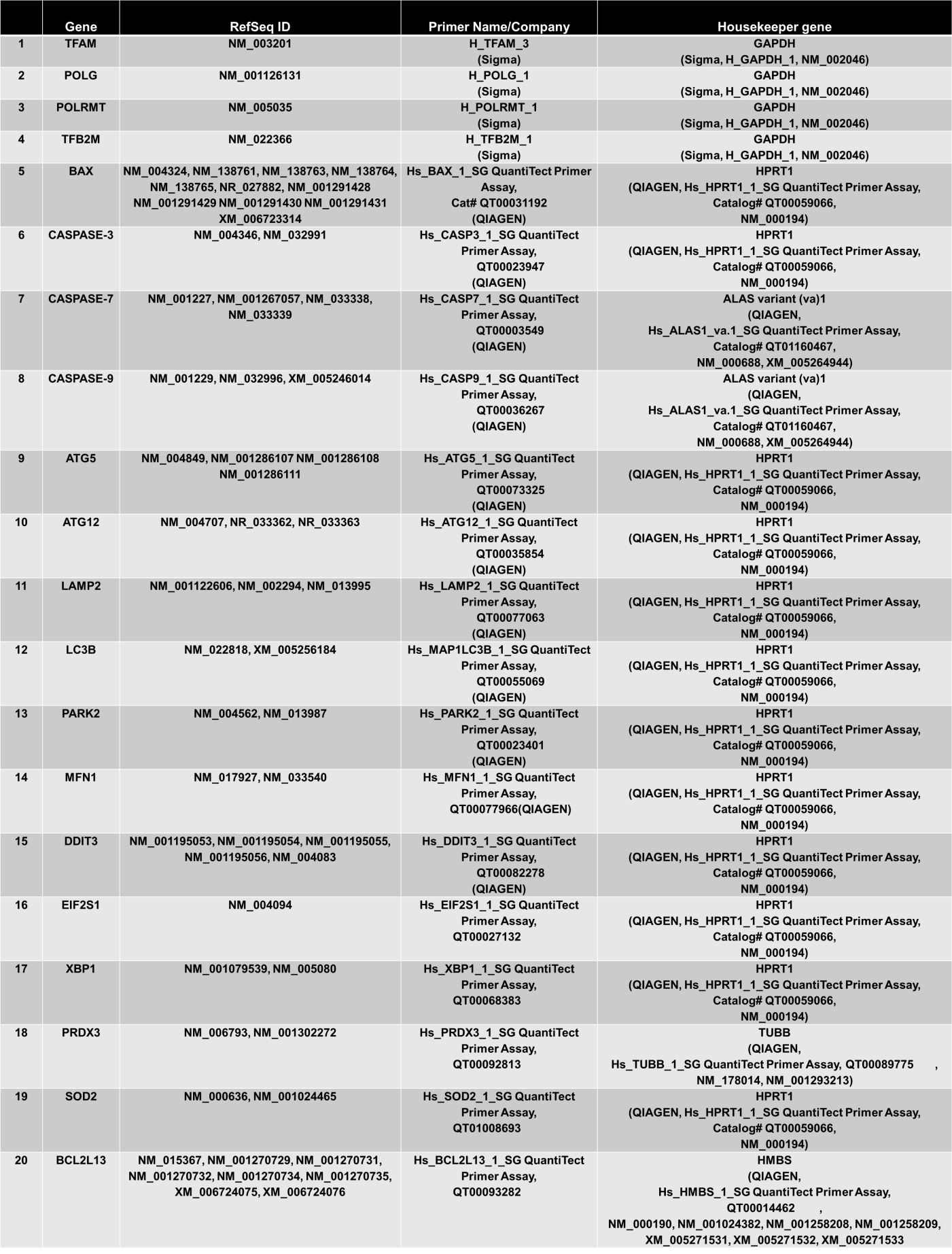
**

**Table S3. List of Antibodies**

**
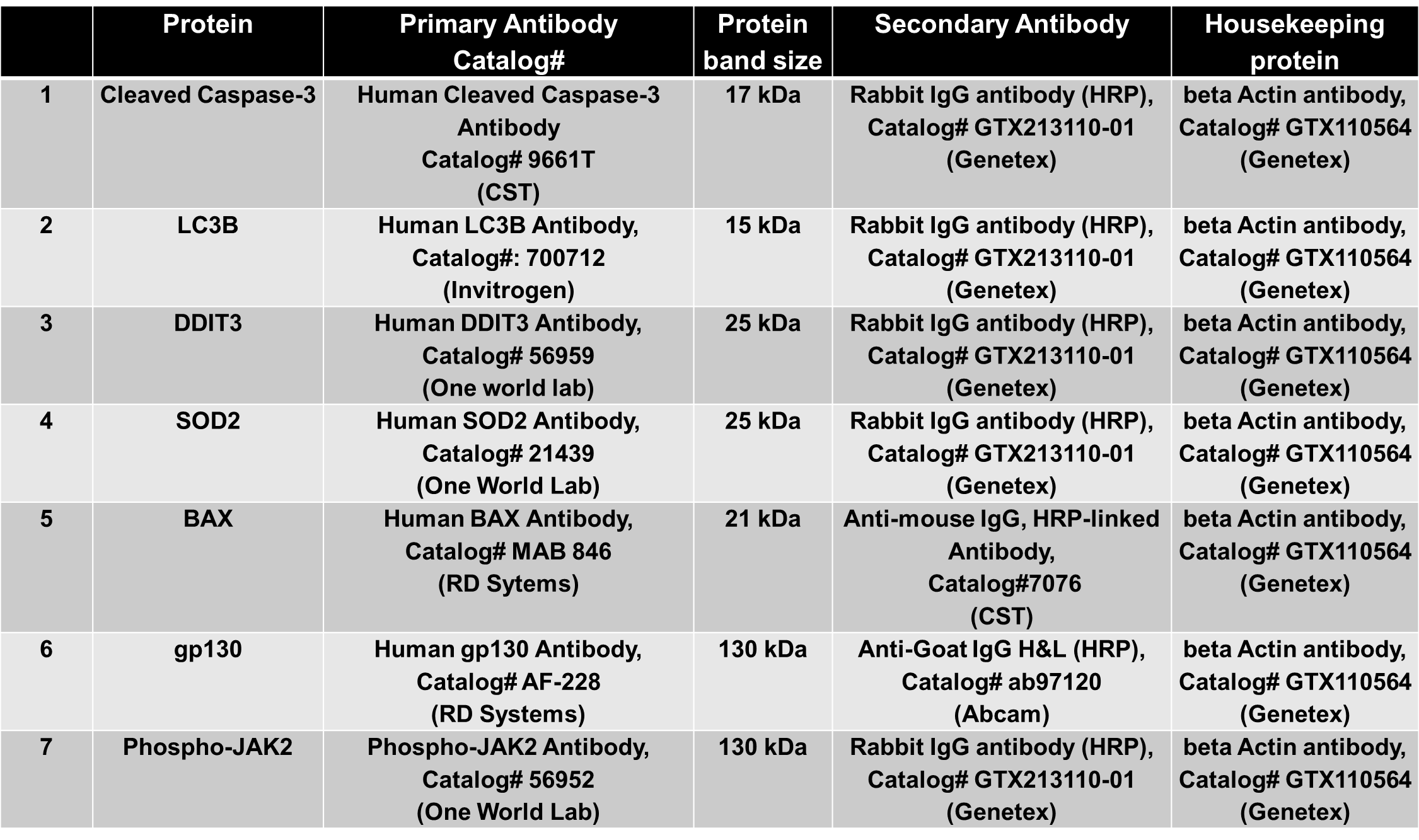
**

**Table S4. AMD cybrids show decreased cell viability and increased mtROS production – results of MTT assay, Trypan Blue assay, and MitoSOX assay**

**
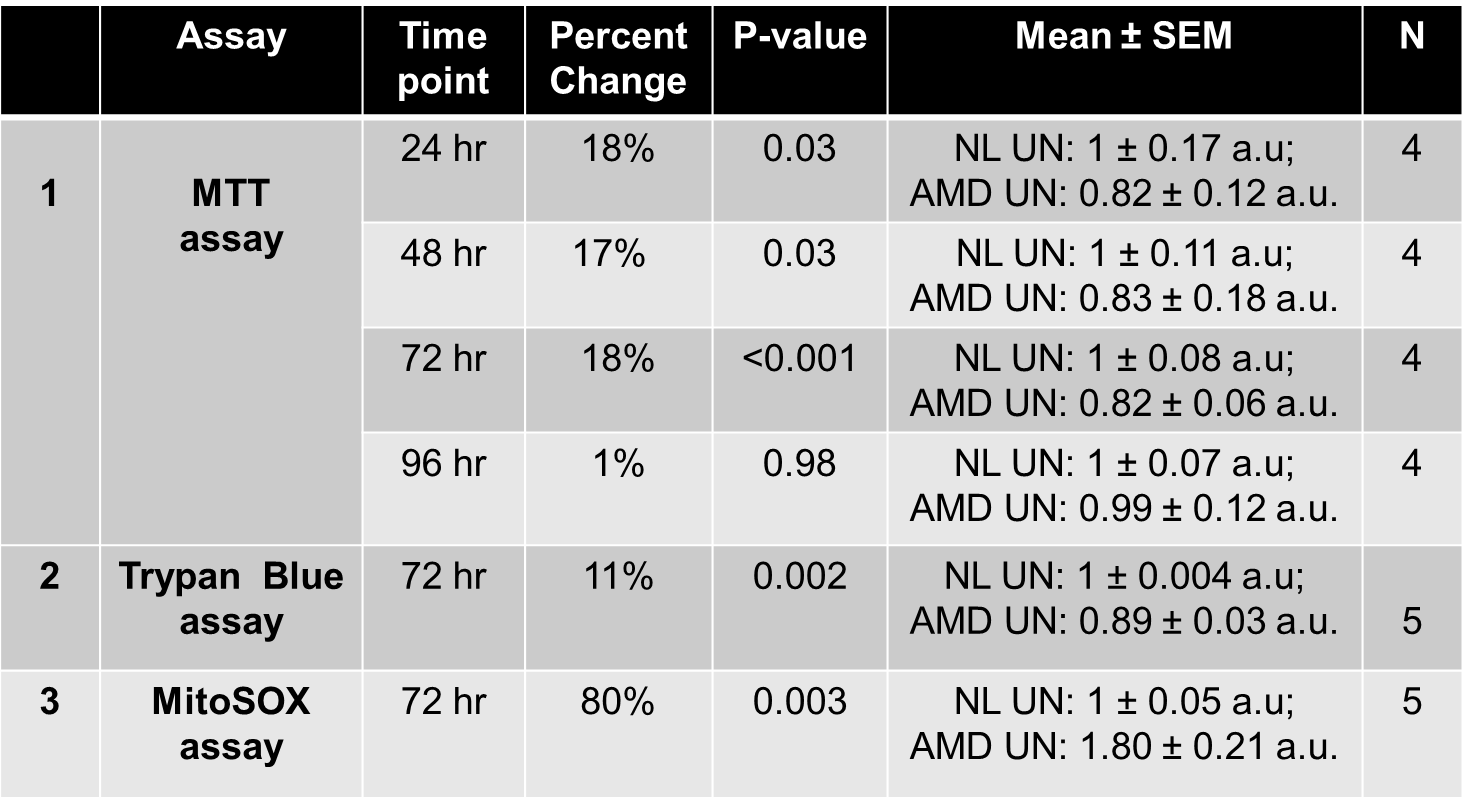
**

**Table S5. Gene Expression data**

**
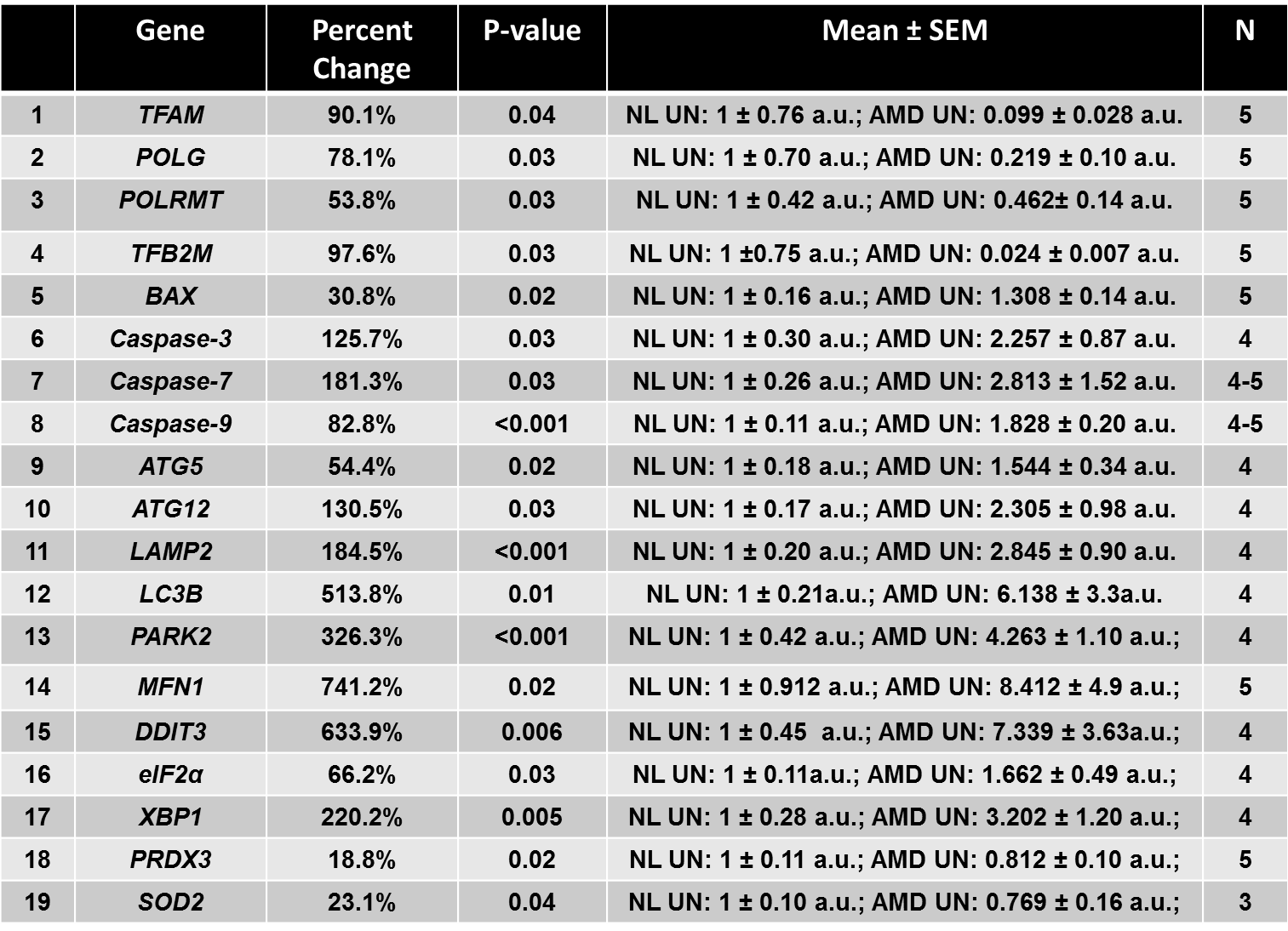
**

**Table S6. Protein levels of apoptosis, autophagy, ER stress, and antioxidant markers**

**
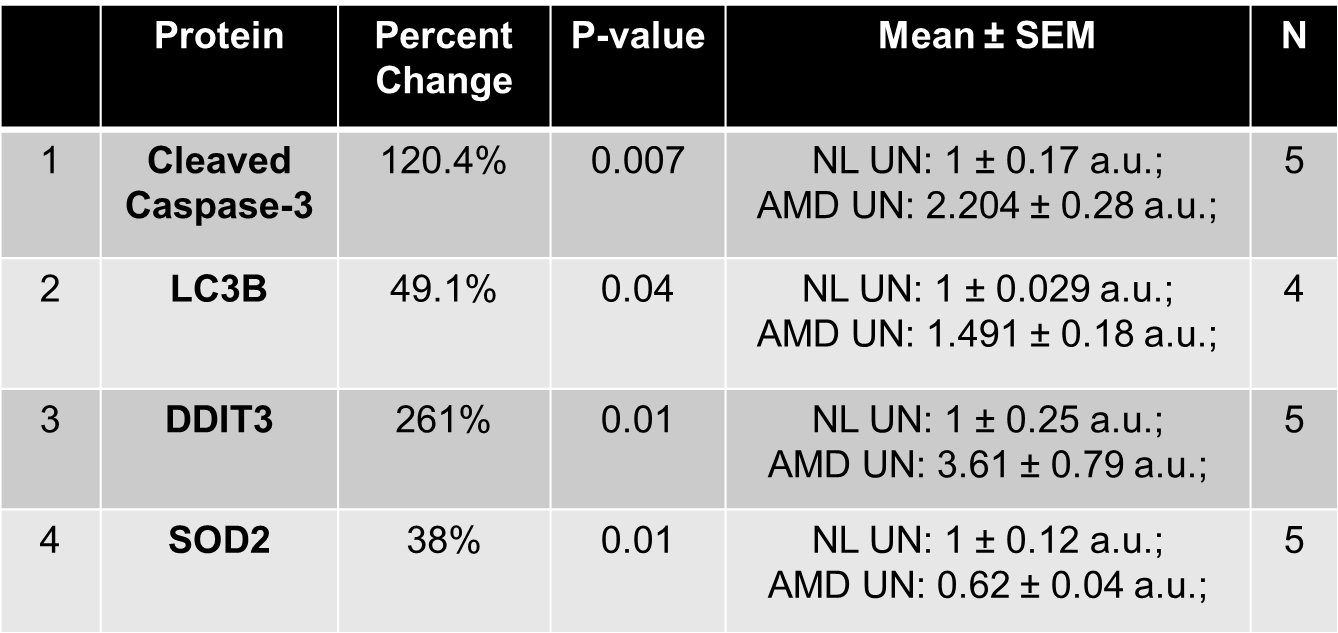
**

**Table S7. Effect of HNG on numbers of apoptotic and live cells in AMD cybrids – YO-PRO/PI staining and Flow cytometry**

**
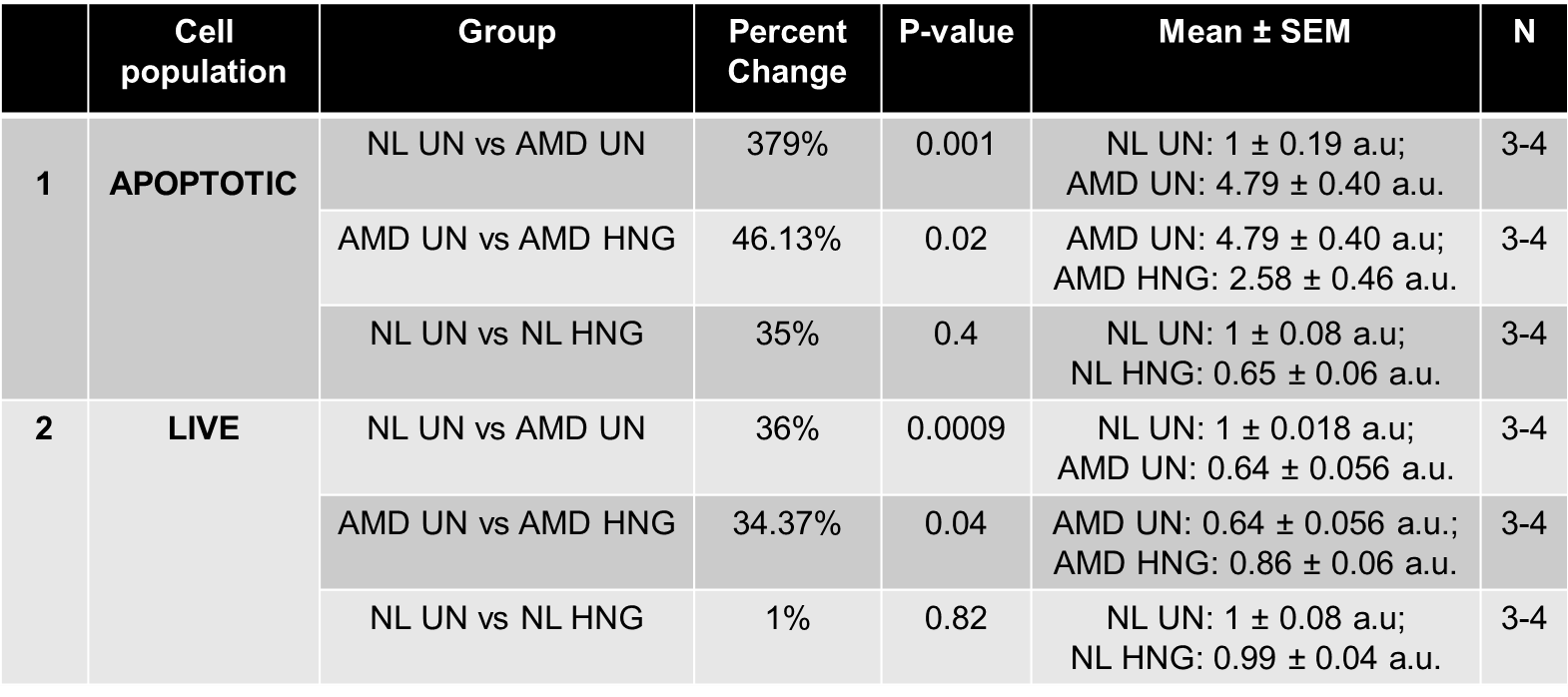
**

**Table S8. Effect of HNG on expression of apoptosis genes: AMD-untreated and AMD HNG-treated cybrids**

**
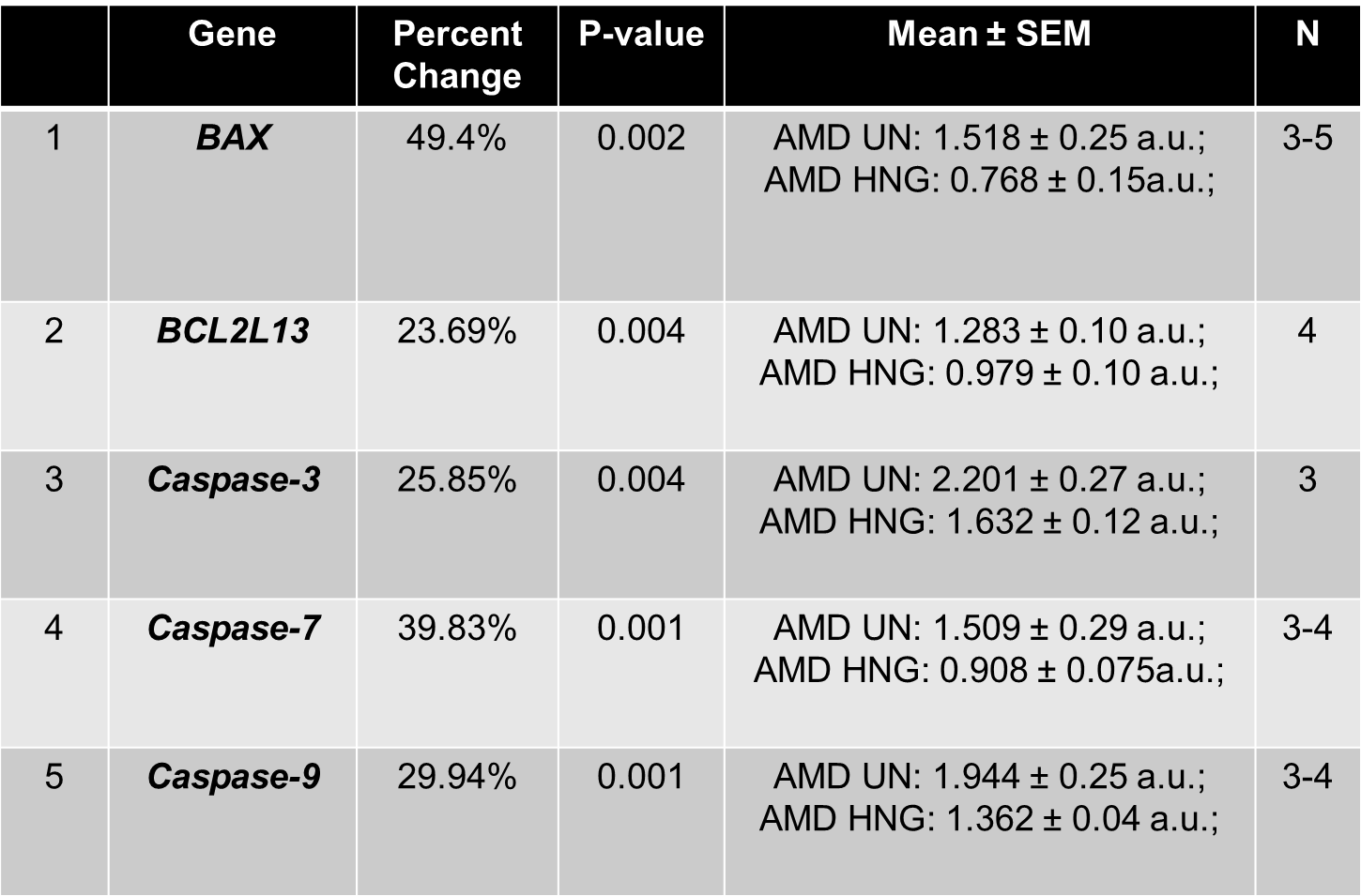
**

**Table S9. Effect of HNG on protein levels of BAX, gp130, and Phospho-JAK2**

**
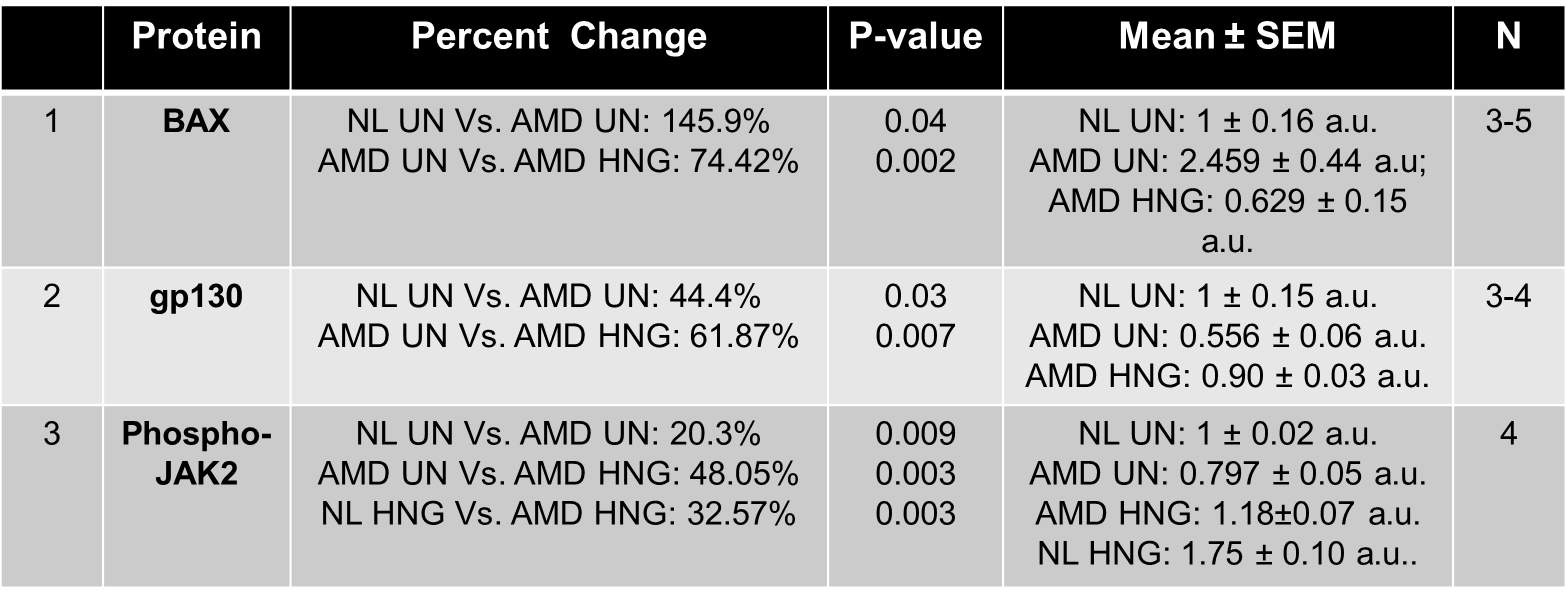
**

**Table S10 A. Effect of HNG on amyloid-β-treated normal cybrids**

**
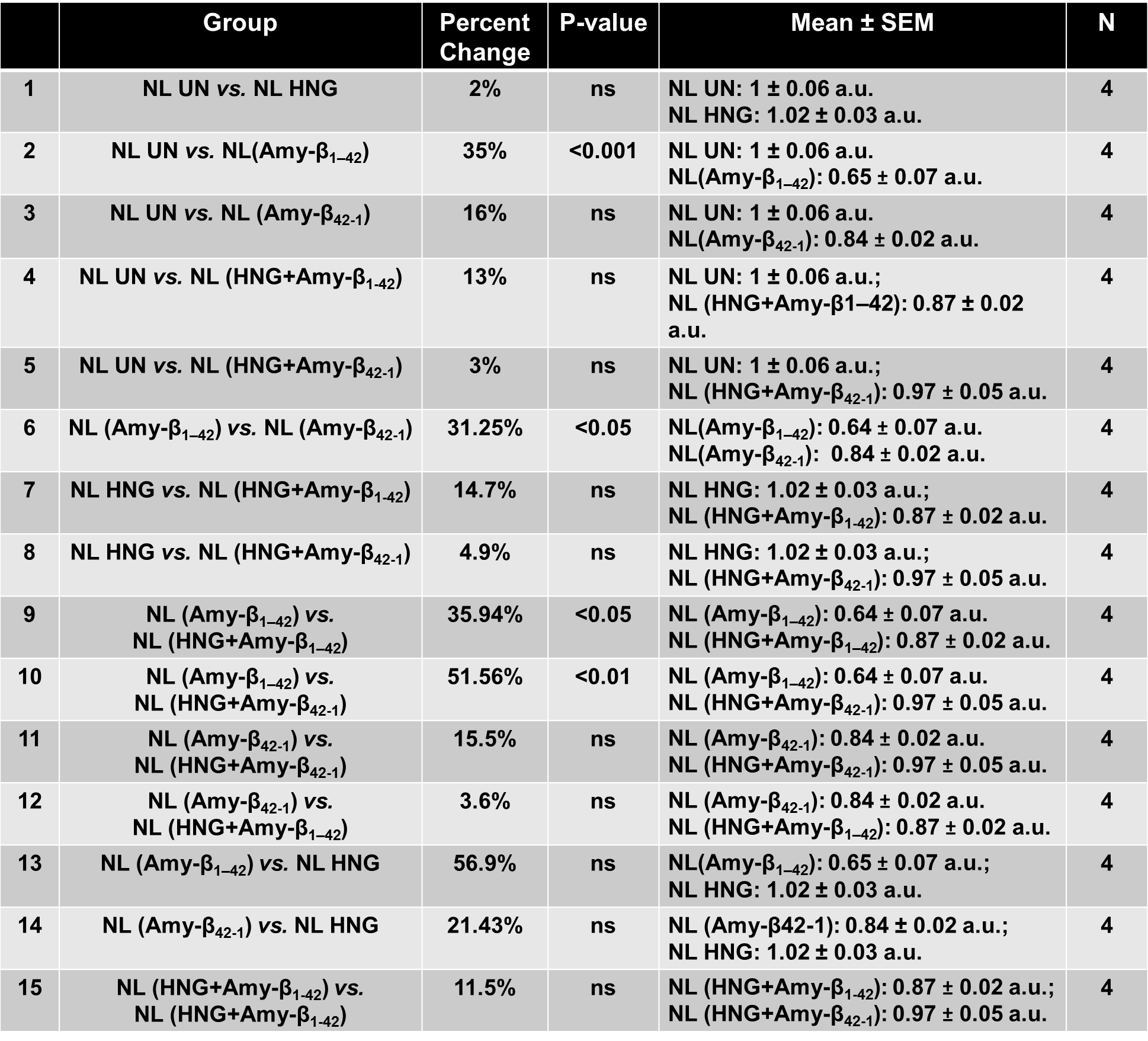
**

**Table S10 B. Effect of HNG on amyloid-β-treated AMD cybrids**

**
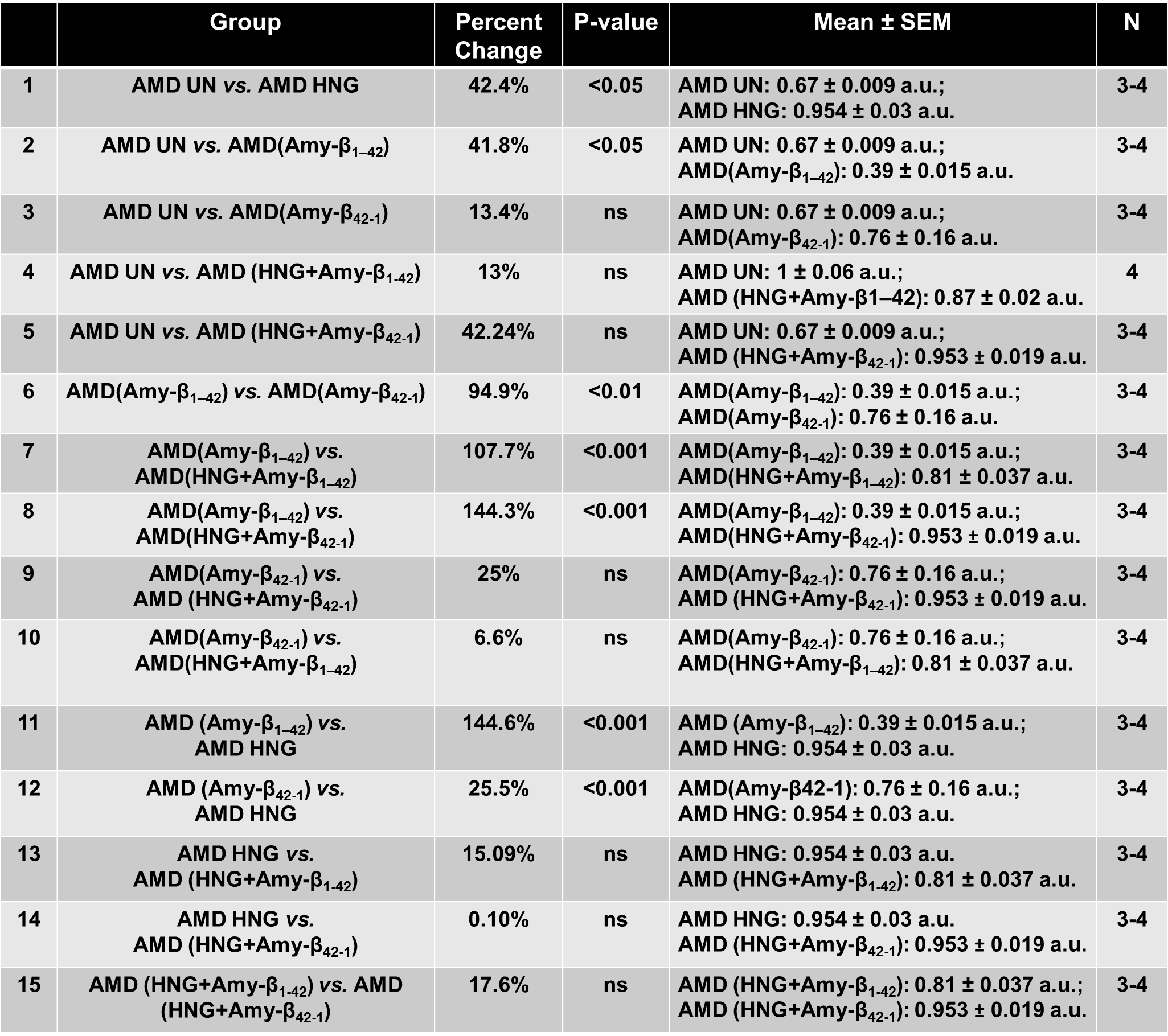
**

**Table S11. HNG enhanced mtGFP fluorescence in AMD cybrids**

**
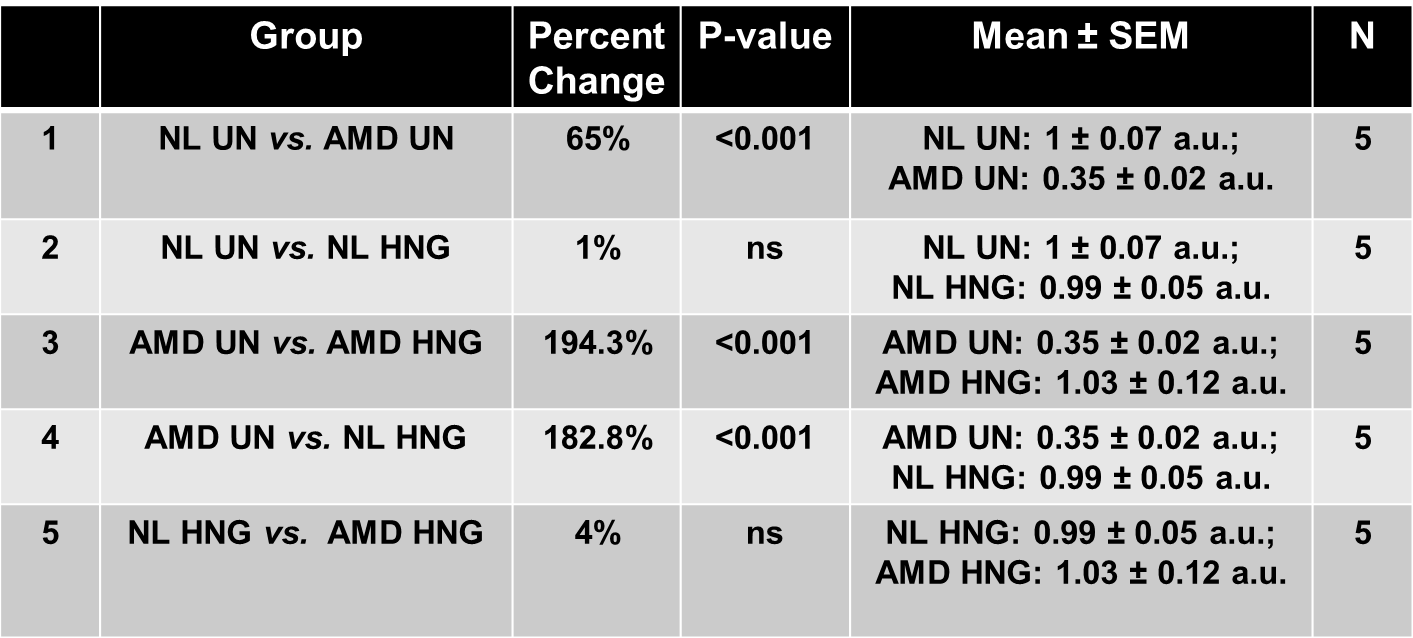
**
